# Navigating complexity: A guide and tools for interdisciplinary research on lower-carbon practices

**DOI:** 10.1007/s13280-026-02369-z

**Published:** 2026-03-17

**Authors:** Sarah J. Boddington

**Affiliations:** Crawford School of Public Policy, Australian National University, JG Crawford Building #132, Lennox Crossing, Canberra, ACT 2600 Australia

**Keywords:** Behaviour change, Climate change, Everyday practice, Low-carbon, Pro-environmental, Sustainability

## Abstract

Lower-carbon practices are socially complex—something that can challenge traditional disciplinary approaches. Researchers can find single disciplines inadequate in capturing the range of factors affecting how different groups engage with these practices. The present study addresses a critical gap in climate research methodology by providing practical tools that enable researchers to move beyond theoretical silos towards interdisciplinary analysis. It presents several inter-connected contributions: a factor landscape mapping the factors shaping responses to three key lower-carbon practices, a compilation of relevant theoretical approaches, and guidance on interdisciplinary research processes. These contributions enable more nuanced, context-sensitive research that captures the social complexity of lower-carbon practices—a critical step towards supporting effective climate action across diverse social systems.

**Supplementary Information:**

The online version contains supplementary material available at 10.1007/s13280-026-02369-z.

## Introduction

Climate change is a coupled problem where society and the environment affect each other (Barry and Born 2013; Hackmann et al. 2014; Schipper et al. 2021; Soininen et al. 2025). One of the ways in which society contributes to climate change is through higher-carbon practices of eating, travel, and energy use (Ivanova et al. 2020). Addressing climate change therefore requires people adopting and sustaining lower-carbon practices in their daily lives (Ivanova et al. 2020; United Nations Environment Programme 2020).

It is estimated that as much as 65 per cent of greenhouse gas emissions can be directly and indirectly linked to the higher-carbon practices of people in high-income countries (Ivanova et al. 2016, 2020). There are four everyday practices with the highest greenhouse gas emissions and potential for intervention (Ivanova et al. 2020). These are driving a petrol- or diesel-fuelled car; household energy fuelled by gas and coal; aviation; and diets that include high amounts of meat and dairy. Dozens of lower-carbon practices can replace these; including replacing diesel and petrol vehicles with electric vehicles, increased use of public transport, installing efficient electrical appliances, installing solar panels, long-distance buses and trains, and lower-meat diets (Ivanova et al. 2020).

Scholars have argued more attention should be paid to the social complexity of transitions to lower-carbon practices (Castree et al. 2014; Hackmann et al. 2014; Pearson et al. 2016; Schipper et al. 2021). Existing practices are ways in which people express their culture, identity, and care for others (Kurz et al. 2020; Böhme et al. 2022; Patterson 2022). Changes in practices can be experienced differently by groups, based on their interests, and experiences of the practice, and their perception of who benefits (Patterson 2022). The differing levels of power, vulnerability, and inequality amongst peoples means some changes to lower-carbon practices benefit some and compound the disadvantage of others (Castree et al. 2014; Dwarkasing 2023). Interventions to change practices can be framed as being for climate change or for other social goals, invoking support, opposition, or indifference depending on people’s group affiliations, and people’s differing priorities and ideas of social progress (Castree et al. 2014; Hackmann et al. 2014). Transitions to lower-carbon practices also involve many actors, meaning that different actors can be mobilised, and mobilise others, in support or opposition to change (Meadowcroft and Rosenbloom 2023; Soininen et al. 2025). The cumulative effect of this social complexity is that interventions can have unpredictable and nonlinear impacts and affect people and groups differently (Bingley et al. 2023; Meadowcroft and Rosenbloom 2023), making it essential that scholars study different contexts, and groups within contexts, to understand how responses to lower-carbon practices vary across societies (Schipper et al. 2021).

It has long been suggested that engaging in interdisciplinarity is one way to grapple with this social complexity (Barry and Born 2013; Castree et al. 2014). In recognition that interdisciplinarity can be challenging (Hazard et al. 2019; Sulik et al. 2025), interdisciplinary processes have been developed for individuals or teams of scholars to follow (Newell 2006; Repko 2006; Menken and Keestra 2016). These emphasise the need to understand a research problem deeply and carefully consider which disciplines may be helpful in studying the problem. Considering a wide range of disciplines is particularly important, because interdisciplinary examination of climate change has been critiqued for drawing on too narrow a set of theories, failing to engage with the “full range of values, means, and ends that might guide human responses” (Castree et al. 2014, 4).

The present study aims to assist scholars in engaging with this social complexity, through providing a guide to interdisciplinary processes and two tools for interdisciplinary exploration of lower-carbon practices. It begins in the “Interdisciplinary processes” section with an introduction to interdisciplinary processes. The “Methods” section outlines the methods used to produce two tools for interdisciplinary exploration of lower-carbon practices. The “Results” section presents the results: the first tool, the factor landscape, aids exploration of research problems. The second tool, the most used theories to study three lower-carbon practices, aids in identifying which disciplines are relevant. The “A guide to using these tools in the research process” section returns to the discussion of interdisciplinarity with a guide to using these tools in interdisciplinary processes. The “Discussion” section discusses implications and limitations. Together, these contributions equip researchers to engage with the social complexity of lower-carbon practices.

## Interdisciplinary processes

Different disciplines emphasise different aspects of the social complexity of lower-carbon practices, with no one discipline representing them wholly (Castree et al. 2014). It is therefore argued that multiple disciplines are needed to understand complex problems and to foster holistic responses (Barry and Born 2013; Castree et al. 2014; Schipper et al. 2021). Interdisciplinarity can be understood as a mode of research that brings theories and methods from two or more disciplines to bear on a particular research problem (Barry and Born 2013; Pohl et al. 2021).

Practitioners can engage in interdisciplinarity as individual scholars or as part of a collaboration of scholars (Repko et al. 2012; Menken and Keestra 2016). Individual interdisciplinary research requires that scholars develop adequacy in each of the relevant disciplines, including a working knowledge of their theories and key concepts (Newell 2006; Repko 2006). Collaborative interdisciplinary research draws on the disciplinary expertise of its team members, but depends on team-based processes, including reflection and dialogue, to build an understanding of how the disciplines are similar and different and to challenge assumptions that underlie the power of some disciplines over others (Menken and Keestra 2016; Hazard et al. 2019; Moon et al. 2021).

**Fig. 1:**
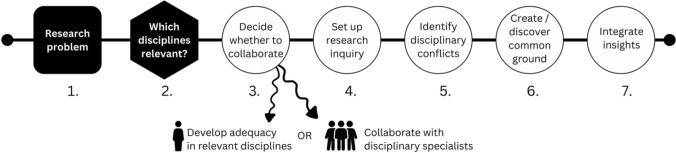
The research process—interdisciplinary at the outset

Scholars of interdisciplinarity have generated interdisciplinary processes—guides for individual and teams of scholars to follow. These processes include Newell’s (2006) steps in the interdisciplinary research process, Repko’s (2006) integrated approach to the interdisciplinary process, both suitable for individuals, and Menken and Keestra’s (2016) IIS model of interdisciplinary research, designed for collaborations. A simplified version, based on these, is shown in Fig. 1.

Research can be interdisciplinary at the outset, or start as a disciplinary inquiry and become interdisciplinary. The first pathway, interdisciplinary at the outset, is shown in Fig. 1 and starts with Step 1, defining and analysing the research problem and Step 2, determining which disciplines are relevant to the research problem. Step 2 can iteratively inform Step 1 as the way different disciplines relate to a problem also helps illuminate different aspects (Newell 2006; Castree et al. 2014). At Step 3, the scholar faces a choice between conducting their inquiry individually or through a collaboration. Step 4 is setting up a research inquiry, which, for collaborative inquiries, can be pursued through separate disciplinary streams or a sequential program of research, where each study informs the next (Hofmann et al. 2025). Whether individual or collaborative, Steps 5, 6, and 7 are about bringing together the insights from the inquiry, a process often described as *integration*. Integration can involve *adding,* using one discipline to extend another discipline, *adjusting* theories or methods in one discipline using another discipline, or *connecting* disciplines around a central idea (Menken and Keestra 2016). There are also alternatives to integration, including *weaving*—keeping disciplinary insights separate and highlighting convergence and contradictions between them (Pohl et al. 2021). Whichever they use, scholars must argue the combining of theories is epistemologically compatible (Sovacool and Hess 2017), or make clear which epistemic assumptions dominate (Barry and Born 2013).

**Fig. 2:**
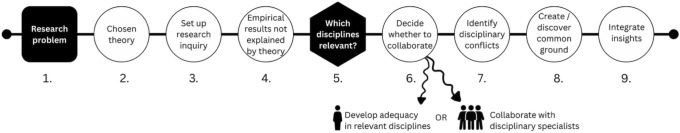
The research process—disciplinary inquiries that become interdisciplinary

The second pathway, disciplinary inquiries that become interdisciplinary, is shown in Fig. 2. In this pathway, at Step 4, a trigger for becoming interdisciplinary might be when a scholar realises the empirical results are not adequately explained by the chosen theory, leading them to inquire into other options for the best ‘theoretical fit’, or explanation, for the data (Sovacool and Hess 2017; Fell et al. 2022).

### Need for tools

Step 1 of both pathways is to define and analyse the research problem. Here, it is useful to know what past studies have found about the topic. There have been compilations of factors shaping practices (Darnton and Horne 2013; Hampton and Adams 2018; Koide et al. 2021; Kamali Saraji and Streimikiene 2023; Albarracín et al. 2024), but none of these have been systematic, cross-disciplinary, and specific to lower-carbon practices. For example, one compilation of factors (Darnton and Horne 2013) was for practices generally, but there are likely differences between what shapes behaviours generally and lower-carbon practices specifically with *pro-environmental motivations* and *knowledge of a practice’s climate/environmental impact* relevant to lower-carbon practices (Bouman et al. 2020). The present study addresses this gap through developing a tool, the factor landscape, which compiles factors shaping three lower-carbon practices.

In the pathways, Step 2 (interdisciplinary at the outset) and Step 5 (becoming interdisciplinary) are deciding which disciplines are relevant. It can be helpful to know which disciplines and theories have been used to study a research problem before. Again, when it comes to lower-carbon practices, past compilations of theories have focused on a particular discipline or a related topic, rather than been cross-disciplinary *and* specific to lower-carbon practices. These include compilations of theories relevant to socio-technical change (Sovacool and Hess 2017), the politics and policy of lower-carbon practices (Roberts et al. 2018; Sovacool and Brisbois 2019), and the psychology of lower-carbon practices (Van Valkengoed et al. 2022). The present study also addresses this gap through developing a second tool, the most used theories in studying three lower-carbon practices.

## Methods

This section outlines the methods used to develop two tools to assist in interdisciplinary exploration of lower-carbon practices. This paper uses a systematic approach, but is not a classic systematic review in the sense that it is aimed at tool development rather than bringing together “all known knowledge on a topic area” (Grant and Booth 2009, 102).

The first stage was identifying previous systematic and meta-reviews relevant to a change from higher- to lower-carbon practices in everyday transport, residential energy, food, and aviation, in high-income liberal-democratic countries. Reviews were chosen as the source material because there are multiple high-quality reviews relevant to lower-carbon practices already published and using primary research would be unmanageable given the quantity of published research for lower-carbon practices. High-income liberal democratic countries are a priority site for change given their relatively higher contribution to greenhouse gas emissions from higher-carbon practices (United Nations Environment Programme 2020). The nature of the challenge is likely different to lower-income countries because practices in high-income countries are likely to be entwined with meanings of “progress” or “affluence” in particular ways (United Nations Environment Programme 2020, 63). Additionally, liberal democratic countries likely differ in important respects from autocratic or non-democratic countries in the process of change, resulting in differences in the extent of climate policy-making (Böhmelt et al. 2016; Lindvall and Karlsson 2024).

The search terms are shown in Box 1. Although the search was cross-disciplinary, the terms “pro-environmental” and “behaviour” are common in psychology, the term “practice” is common in sociology, and the terms “intervention” and “determinant” are common in health research, and so it was expected more of the results would be from those disciplines.

**Box 1:** Search terms

(TITLE (“Systematic review” OR “Meta review” OR “Meta synthesis” OR “Meta analysis”) AND TITLE-ABS-KEY (“Low carbon” OR “pro-environmental” OR “net-zero”) AND (practice OR behavior) OR TITLE-ABS-KEY (change OR intervention OR shift OR determinant OR reduction) AND (transport OR bike OR bicycle* OR vegan OR vegetarian OR “plant-based” OR transport OR “electric vehicle” OR walk* OR aviation OR “residential electricity” OR “residential energy” OR solar OR “photovoltaic”) AND PUBYEAR > 2015	

**Fig. 3:**
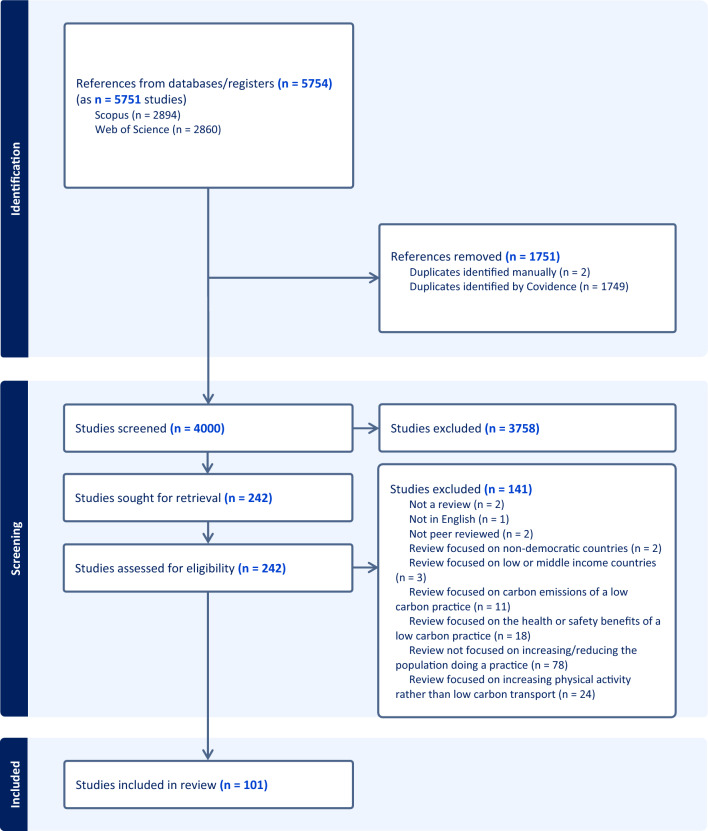
PRISMA diagram outlining the search process to identify relevant reviews on lower-carbon practices

As shown in the PRISMA diagram in Fig. 3, a search of two databases resulted in 5751 articles. The second stage was removing duplicates and screening. The inclusion and exclusion criteria are given in Table S1 of the Supplementary Materials.

Screening resulted in 101 articles covering three lower-carbon practices: everyday transport (60), residential energy (11), and food (12), and multi-practice studies that include at least one of these three areas (18). The lack of reviews on aviation meant this lower-carbon practice was excluded from the analysis. The third stage was analysing the 101 articles using an extraction form created in Covidence (Kellermeyer et al. 2018).

Coding of factors followed a two-stage inductive process (Sect. 2 Supplementary Materials); this produced 25 factors. Coding of theories was done through identifying theories, models or frameworks (called “theories” for short) mentioned in the reviews, producing 151 theories in total, with 45 theories mentioned more than once (Table S3 Supplementary Materials). These were short-listed if they were mentioned in relation to at least two out of the three practices, at least four times across all reviews, resulting in 15 theories.

## Results

### The factor landscape

The first tool from this systematic approach is the factor landscape, a compilation of factors identified in reviews as shaping how people engage with three lower-carbon practices. Analysis of 101 reviews identified 25 factors, as set out in Table 1.

**Table 1:** Factors identified as shaping the lower-carbon practices of everyday transport, residential energy, and food

Number	Factor
1	Cost/financial assistance
2	Non-financial assistance or policies
3	Safety
4	Subjective norms
5	Environmental motivation
6	Knowledge of climate/environment impact
7	Other motivation, e.g. health, saving money
8	Social norms
9	Narratives
10	Life events
11	Social networks
12	Culture
13	Family dynamics
14	Social support
15	Sensory factors
16	Behavioural control
17	Emotional responses
18	Politics and interests
19	Infrastructure/built environment
20	New technologies
21	Services
22	Knowledge of the low-carbon practice
23	Skills
24	Ease of the practice
25	Routines and habits

**Fig. 4:**
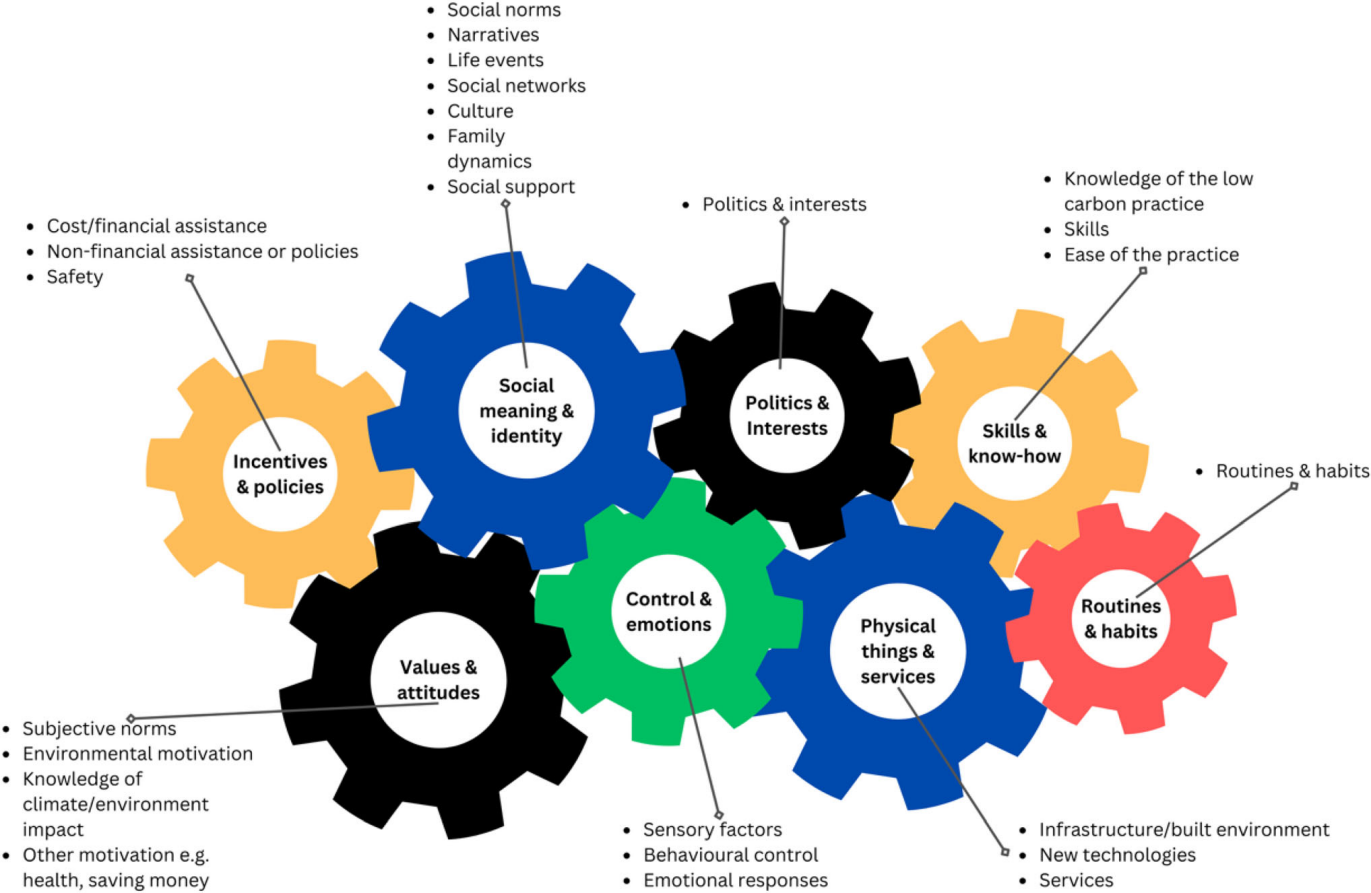
The factor landscape: factors identified in previous studies as shaping people’s responses to three lower-carbon practices, sorted into eight clusters

The factor landscape is presented in Fig. 4, showing these 25 factors sorted into eight clusters, with each cluster linking together conceptually related factors: incentives and policies; values and attitudes; social meaning and identity; control and emotions; politics and interests; physical things and services; routines and habits; and skills and know-how. The clustering is provided to organise this complexity, but it is done with the acknowledgment that this is only one of many possible ways to group these factors. There are multiple other potential ways to group them because each of these factors is in and of themselves multifaceted concepts that interact and shape each other. For example, culture (factor 12) can be regarded as an “ideational subsystem within a vastly complex system, biological, social and symbolic” (Keesing 1974, 94). It is therefore a meta-concept that encompasses other factors including emotional responses (factor 17), behavioural control (factor 16), and social norms (factor 8). As is discussed in “ the A guide to using these tools in the research process” section, the factor landscape is intended to enable exploration of these linkages between factors (Fig. 4).

### Most used theories

The second tool from this systematic approach is a list of the most used theories used to explore three lower-carbon practices. The 15 most used theories are set out in Table 2. As expected, this collection of theories are predominantly from the disciplines of sociology and psychology, but also include some from economics, science and technology studies, and political science.

**Table 2:** The most used theories for three lower-carbon practices, by discipline and level of analysis. ^a^T, Everyday transport; E, Residential energy; F, Food

Rank of prevalence of mentions	Theory	Dominant discipline	Level of analysis	Which practice^a^
1	Theory of planned behaviour	Psychology	Micro	T, E, F
2	Social-ecological framework	Sociology	Macro	E, F
3	Social identity approach	Psychology	Meso	T, E, F
4	Value-belief-norm	Psychology/sociology	Micro	T, E
5	Norm activation model	Psychology	Micro/Meso	T, E, F
6	Social cognitive theory	Psychology	Micro	T, E
7	Diffusion of innovation	Sociology/science and technology studies	Meso	T, E
8	Focus theory of normative conduct	Psychology	Micro/Meso	T, E
9	Theory of reasoned action	Psychology	Micro	T, E
10	Behavioural economics	Economics/psychology	Meso	T, E
11	Habit theory	Psychology	Micro	T, E
12	Transtheoretical model of behaviour change	Psychology/public health	Micro	E, F
13	Socio-technical transitions	Science and technology studies/political science	Macro	T, E
14	Social practice theory	Sociology	Meso	T, E
15	Climate justice	Political science/law	Macro	T, E

Table 2 identifies whether the theories are useful for analysis at a macro-, meso-, or micro-level (Geels et al. 2016; Sovacool and Hess 2017). The theory of Socio-technical Transitions can be used for analysis at the systems-change, or macro-level (Sovacool and Hess 2017). The Social Identity Approach is helpful at the meso-level, in understanding how contexts shape practices (Fielding and Hornsey 2016). The Transtheoretical Model of Behaviour Change assists at the micro-level, in understanding how individuals can shift their practices (Friman et al. 2017).

As discussed further in  the “A guide to using these tools in the research process” section, it is helpful to not only know which theories within which disciplines may be relevant to a problem, but also to have a working knowledge of key concepts within that theory, and how that theory explains the problem (Newell 2006; Repko 2006; Fell et al. 2022). A working knowledge of theory is needed even in collaborative teams, as building understanding of each other’s disciplines is key to an effective collaboration (Menken and Keestra 2016). To assist scholars in beginning this process of learning about the theories relevant to three lower-carbon practices, Table 3 provides a summary of each theory and maps them against the 25 factors, highlighting which theories offer explanations for which factors.

**Table 3:** Short description of the 15 most used theories, mapped against the 25 factors

Theory	Short description, with examples of where a review mentions a factor important in the theory	This theory focuses on the following of the 25 factors
Theory of planned behaviour	In the Theory of Planned Behaviour, practices are thought to be shaped by peoples’ perceptions they have the material resources, time and capacity to adopt the new practice, known as their ‘perceived behavioural control’ (Ajzen 1991; Bamberg 2013), which can be technology specific (Scheller 2024). Other elements of the theory are a person’s ‘attitude’ towards the practice and ‘social norms’ (called ‘subjective norms’ by Ajzen (1991)); a person’s attitude is shaped by the perceived benefits of the practice, including environmental and financial benefits (Scheller 2024). Reviews in this study found behavioural control to be a strong predictor of a person engaging in lower-carbon transport (Hoffmann et al. 2017; Lanzini and Khan 2017) and to an extent in green energy behaviour (Hu et al. 2020)	**Values & attitudes**: Environmental motivation; Other motivations **Social meaning & identity**: Social norms **Control & emotions**: Behavioural control **Physical things & services**: New technologies
Social-ecological framework	Often shown as part of nested systems, Social-ecological Frameworks explore a broad range of factors shaping practices (Sallis et al. 2006). They include the built environment and technology, the natural environment, the information environment, the policy environment (land use zoning, financial incentives, transport investment), the socio-cultural environment (advocacy, crime, social support, intrapersonal modelling), the perceived environment (safety, attractiveness, comfort, convenience), and intrapersonal factors (family dynamics, psychology, demographics) (Sallis et al. 2006). Researchers using this approach also consider how factors shape practices differently for different groups in society, including based on age, disability and ethnicity (Steinbach et al. 2011; Lindsay and Lamptey 2019). One review in this study (Taufik et al. 2019) listed the individual, intrapersonal, and environmental factors shaping consumption of lower-carbon food; for example, environmental determinants include its taste and texture, packaging, portion size, availability, pricing, the eating environment, and food outlets	**Incentives & policies**: Cost/financial assistance; Non-financial assistance or policies; Safety **Social meaning & identity**: Social norms; Narratives; Life events; Social networks; Culture; Family dynamics; Social support **Physical things & services**: Infrastructure/built environment; New technologies; Services **Skills & know-how**: Knowledge of low-carbon practice; Skills; Ease of the practice
Social identity approach	In the Social Identity Approach, it is thought that peoples’ multiple social identities guide their practices, depending on how strongly they identify, and the salience of that identity in a context (Tajfel 1981; Turner et al. 1987; Fielding and Hornsey 2016; Spears 2021). In some contexts, subjective norms (called personal norms in this theory) may be more relevant (Turner et al. 2006). Social networks and social identities can normalise higher-carbon practices, but they can also be sites where lower-carbon practices originate or are fostered. For example, those who identify with their neighbourhood are more likely to follow their neighbours in installing solar panels, or match their energy use to those of their neighbours (Graziano and Gillingham 2015; De Dominicis et al. 2019). Emotion is an important part of the process of identifying with a group (Spears 2021). One review in this study (Vesely et al. 2021) found social identity can be a strong predictor of climate-friendly intention and behaviour when the group is clearly linked to climate issues and action (e.g. through supportive group norms)	**Values & attitudes**: Subjective norms **Social meaning & identity**: Social norms; Social networks; Culture **Control & emotions**: Emotional responses
Value-belief-norm	In the Value-Belief-Norm model, subjective norms (called personal norms in this theory) are considered to shape engagement in practices (Stern et al. 1999; Stern 2000). Elements that contribute to subjective norms include values, an ecological worldview, an awareness of consequences, and ascription of responsibility (Stern et al. 1999; Lind et al. 2015). One review in this study (Lanzini and Khan 2017) found that personal and social norms were good predictors of travel mode intention, but not practice	**Values & attitudes**: Subjective norms; Environmental motivation; Knowledge of climate/environmental consequences
Norm activation model	In the Norm Activation Model, subjective norms (called personal norms in this theory) are considered to shape intentions to engage in practices (Schwartz 1977; De Groot and Steg 2009; Van Der Werff and Steg 2015). Elements that contribute to subjective norms include an awareness of consequences and ascription of responsibility. At times people might avoid feeling responsible for adverse consequences, for example through assigning responsibility elsewhere or denying their actions can have an impact (Klöckner and Matthies 2004). One review in this study (Hoffmann et al. 2017) found that personal intentions and habit generated larger effect sizes than subjective norms when it came to travel mode choice	**Values & attitudes**: Subjective norms; Environmental motivation; Knowledge of climate/environmental impact
Social cognitive theory	In Social Cognitive Theory, it is thought practices result from personal and social factors that interact with institutional systems (Bandura 1999). Key personal factors are personal self-efficacy, the sense that people feel they can achieve their aims; proficient performance, once a person knows a practice they no longer need to exert a high level of cognitive control; emergent interactive agency, the idea that people are producers of practices through reflection and creativity; and subjective norms, or internalised regulatory standards (called moral reasoning in this theory). Some of the key social factors are: symbolic modelling, a way of conveying new practices to others; influencing through social networks; social meaning communicated through symbols; and collective efficacy, an assessment about what can be collectively achieved. One review in this study (Chatzigeorgiou and Andreou 2021) found digital feedback reduced energy consumption, and goal setting and gamification offered promising results	**Values & attitudes**: Subjective norms **Social meaning & identity:** Social norms; Social networks; Culture **Control & emotions**: Behavioural control **Values & attitudes:** Environmental motivation; Other motivation **Routines & habits**
Diffusion of innovation	Some lower-carbon practices involve new technologies, such as induction cooktops, heat pumps, electric vehicles, electric scooters, and new plant-based proteins. Diffusion of Innovation Theory is used to explain the spread of new technologies (Rogers 1995; Dearing 2009). Researchers using this theory focus on new technologies’ relative advantage; its savings in time and effort, decrease in discomfort, and cost savings, the impact of monetary and non-monetary incentives, its compatibility with values, beliefs, norms, culture, and needs; the technology’s complexity; its trialability; and its observability (Rogers 1995; Dearing 2009). They often focus on the characteristics of early adopters of new technologies as well as the spread of a technology within society through word of mouth, media, and social networks (Centola et al. 2018; Horvat et al. 2020). One review in this study (Kastner and Stern 2015) found factors such as operational comfort, cost and whether a person has tried the technology before shapes whether they want to buy an energy-efficient heating system. Another review looking at electric vehicles found early adopters to be motivated by the environment or futurism (Daramy-Williams et al. 2019)	**Incentives & policies**: Cost/financial assistance; Non-financial assistance or policies **Values & attitudes**: Environmental motivation; Other motivation **Social meaning & identity**: Social networks; Culture; Social support **Physical things & services**: Infrastructure/built environment; New technologies; Services **Routines & habits** **Skills & know-how**: Knowledge of low-carbon practice; Skills; Ease of the practice
Focus theory of normative conduct	In the Focus Theory of Normative Conduct, it is thought the practices of others can be influential (Cialdini et al. 1991; Miller and Prentice 2016). Social norms are the practices most people within a relevant social group do (descriptive norms) or think should be done (injunctive norms) (Cialdini et al. 1991; Cialdini and Goldstein 2004). Social norm influence is exerted through social networks and social identities (Centola 2021; Spears 2021). People’s practices can also be affected by their subjective norms (called personal norms in this theory). Norms only shape practices when they are salient. One review in this study (Bergquist et al. 2023) found people’s engagement in a range of lower-carbon practices are affected by the practices and opinions of others, and therefore, interventions based on social comparisons were amongst the most effective	**Values & attitudes**: Subjective norms **Social meaning & identity**: Social norms; Social networks
Theory of reasoned action	Like the Theory of Planned Behaviour, the important elements of the Theory of Reasoned Action (Fishbein and Ajzen 2009) are a person’s ‘perceived behavioural control’ (based on beliefs about the personal and environmental factors affecting the practice) and their ‘attitude’ towards the practice (based on whether they think it will be positive or negative for them). This theory also puts weight on ‘social norms’ (called ‘perceived norms’). Differently, Fishbein and Ajzen (2009) acknowledge that a wide variety of factors shape a person’s perceived behavioural control, attitude, and social norms, including emotions, values, perceived risk, past behaviour, culture, knowledge, and interventions. Also differently, Fishbein and Ajzen (2009) note whether an *intention* is translated into a *practice* depends on a person’s actual control, which is shaped by their skills, abilities, and environmental factors. They also take into account habits: that these factors can be shaped spontaneously, without careful deliberation, when a person is doing a familiar practice (Fishbein and Ajzen 2009). One review in this study (Olsson et al. 2019) discussed how factors such as perceived fuel saving, socialising, and saving money had positive effects on carpooling (these would all be considered to be related to whether a person thinks the practice is positive or negative for them)	**Values & attitudes**: Environmental motivation; Other motivation **Incentives & policies**: Cost/financial assistance; Non-financial assistance or policies; Safety **Social meaning & identity**: Social norms; Culture **Control & emotions**: Behavioural control, emotional responses **Physical things & services**: Infrastructure/built environment; New technologies; Services **Skills & know-how**: Knowledge of low-carbon practice; Skills; Ease of the practice **Routines & habits**
Behavioural economics	Although economic theories are broader, the reviews in this study focused more on Behavioural Economics, which is concerned with how people react to motivations and emotions associated with gain, loss and risk. Behavioural economics draws on knowledge of cognitive biases to highlight the various ways in which people do not make a straightforward calculation of costs and benefits, as is implied by rational actor approaches (Bamberg 2013; Orlove et al. 2020). One review in this study discusses how people often do not do lower-carbon energy retrofits, even when they would save money, because cognitively they give more weight to short-term costs than they do future savings (Lang et al. 2021)	**Incentives & policies**: Cost/financial assistance; Non-financial assistance or policies; Safety **Values & attitudes**: Subjective norms; Environmental motivation; Knowledge of climate/environment impact; Other motivation **Control & emotions**: Emotional responses
Habit theory	Habit Theory explains when practices are done consciously or unconsciously. Habitual actions use System “1” unconscious, associative thinking as opposed to System “2” reflective thinking (Kahneman 2011). Habitual actions save people the cognitive energy and time associated with reflective thinking, but it can be hard for a new practice to break in when people are not making conscious decisions about their practices. Many high-carbon practices are habitual, and unless there are strategies to ‘unfreeze’ the habit, lower-carbon practices are often not considered. Once a person is engaged in a reflective choice, their perceived behavioural control, along with their skills to do the practice, can affect their uptake of a lower-carbon practice. Reviews in this study (Hoffmann et al. 2017; Lanzini and Khan 2017; Piatkowski and Bopp 2021; Valli et al. 2023) found habits important in both transport and food practices	**Routines & habits**
Transtheoretical model of behaviour change	Researchers using the Transtheoretical Model of Behaviour Change consider how to ‘unfreeze’ a habitual practice so that a person can contemplate a different practice (Bamberg and Schulte 2018). They identify supports for the multiple stages of change (Bamberg 2013; Friman et al. 2017, 2019; Bamberg and Schulte 2018). People can get stuck in the process of change without active support from others (Bamberg 2013). ‘Activating’ relevant personal and social norms, trialling lower-carbon practices, goal setting, obstacle reduction, skill improvement, and social reinforcement are all strategies to assist in unfreezing the habit, forming attitudes, and increasing perceived behavioural control (Bamberg 2013; Friman et al. 2017). One review in this study (Foster et al. 2018) looked at interventions to increase walking, some of which were based on the transtheoretical model of behaviour change; it found a combination of approaches had the biggest impact. This included mass media campaigns, community programs such as walking groups and individual advice, and built infrastructure	**Values & attitudes**: Subjective norms; Environmental motivation; Other motivation **Social meaning & identity**: Social norms; Life events; Social networks; Social support **Control & emotions**: Behavioural control **Physical things & services**: Infrastructure/built environment; New technologies; Services **Routines & habits** **Skills & know-how**: Knowledge of low-carbon practice; Skills; Ease of the practice
Socio-technical transitions	The Theory of Socio-technical Transitions can be used to analyse the politics and interests affecting the spread of lower-carbon practices. The focus of this theory is how systems change from a dominant “socio-technical regime” to the widespread dissemination of “niche innovations” (Geels et al. 2017a). Socio-technical transition theorists see change as a process of contestation between coalitions of actors, using the tools of policy, public opinion, discourses, new technologies, and opportunistically seizing upon contextual changes (Geels et al. 2017b; Mylan et al. 2019; Corradi et al. 2023; Dueñas-Ocampo et al. 2023). For example, a coalition between green NGOs, industrial firms and biogas worked together to harness public opinion to block an attempt by fossil fuel interests to end the policy that had made renewable energies cost-effective (Geels et al. 2017b). But current institutional structures and these tools can also be used to protect the status quo. Researchers using this theory understand that limiting and disrupting the “socio-technical” regime is sometimes necessary to enable the spread of “niche innovations”, this can be done through curbing policies (Geels et al. 2017a, b; Van Rijnsoever and Leendertse 2020)	**Incentives & policies**: Cost/financial assistance; Non-financial assistance or policies **Social meaning & identity**: Social norms; Narratives; Social networks; Culture **Politics & interests** **Physical things & services**: Infrastructure/built environment; New technologies; Services
Social practice theory	In Social Practice Theory, practices are shaped by the interaction between social meaning, materials, and competences (Shove et al. 2012). ‘Competences’ encompasses both the skills to do a practice and ‘know-how’, for example how to save time spent on meals, such as buying or cooking in bulk or combining meals with social activities (Hoolohan et al. 2018). Some social practice theorists also add a fourth element of ‘formal rules’; such as policies and government programs (Bartiaux et al. 2014). Practices become routine ways of operating in society. They are reinforced by other practices, for example the usual practice of driving is reinforced by time pressures associated with practices around school and work time (Southerton 2007). Social practice theorists see a role for policy to reshape material and institutional conditions (Shove 2010; Nash et al. 2017; Watson et al. 2020) to create more “‘envirogenic’ environments” conducive to lower-carbon practices (Shove 2010, 1282). Several reviews in this study mention social practice theory (Pollard and Wagnild 2017; Javaid et al. 2020; Scepanovic et al. 2017). One review (Javaid et al. 2020) discusses how material factors interact with social and individual factors in people’s travel mode choice; for example, it finds increased fuel taxes can interact with availability of public transport infrastructure to shape use of public transport	**Incentives & policies**: Cost/financial assistance; Non-financial assistance or policies; Safety **Social meaning & identity**: Social norms; Narratives; Life events; Social networks; Culture; Social support **Physical things & services**: Infrastructure/built environment; New technologies; Services **Routines & habits** **Skills & know-how**: Knowledge of low-carbon practice; Skills; Ease of the practice
Climate justice	Climate Justice, and the associated theory of Environmental Justice, are normative theories, which focus analysis on who is included and excluded from higher- and lower-carbon practices (Martiskainen et al. 2021; Newell et al. 2021; Gulliver et al. 2023). Dwarkasing (2023, 8) notes the starting point for the transition to lower-carbon practices is inequity and all changes have the potential to “weaken or strengthen existing inequalities”. This is because, as transitions occur, people who are already disadvantaged are likely to face unequal access to new lower-carbon practices, and bear increased costs associated with continuing higher-carbon practices (Baker et al. 2018; Tidemann et al. 2019; Martiskainen et al. 2021; Sovacool et al. 2022; Hanke et al. 2023). Climate and environment justice also focuses analysis on who is doing the most and least in contributing to environmental and climate harms, in order to recognise this injustice and create procedures for a rebalancing (Newell et al. 2021; Newell 2022). It also includes a focus on the high-income countries that are responsible for most of the emissions from higher-carbon practices (Ivanova et al. 2020; Chancel 2022). Many reviews in this study note that inequities concentrated according to ethnicity, gender, class and wealth, and geography determine people’s access to lower-carbon transport, heating and food practices (Marzi et al. 2018; Rothman et al. 2018; Scepanovic et al. 2017; Christidis et al. 2021; Ravi et al. 2021; Riazi et al. 2022; Dwarkasing 2023)	**Incentives & policies**: Cost/financial assistance; Non-financial assistance or policies **Politics & interests**

## A guide to using these tools in the research process

The first tool, the factor landscape, and the second tool, the most used theories, assist scholars in their interdisciplinary exploration of one or more of the three lower-carbon practices. Step 1 in both pathways is to define and analyse the research problem. The aim is to identify the major factors of social complexity (Newell 2006), which are shaped by the context and the practice being studied. In analysing the research problem, scholars (Newell 2006; Menken and Keestra 2016, 59) suggest using guiding questions such as “what do I already know about the problem?”, “what aspects…are important to consider?”, “what other problems does it relate to?”, “who is this a problem for?” and “from which perspectives can I look at this problem?”.

**Fig. 5:**
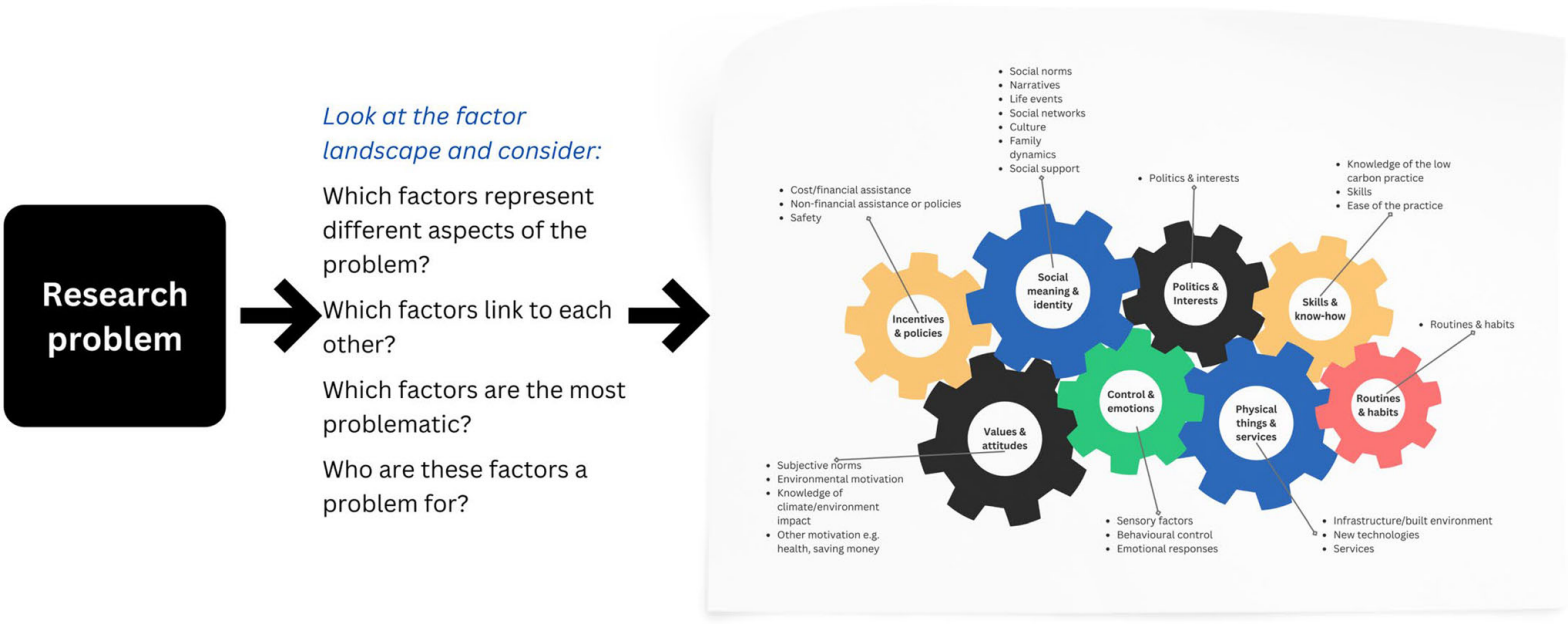
Analysing the research problem using the factor landscape

The first tool, the factor landscape, can help to expand thinking at this stage of research. Scholars can use the factor landscape, as outlined in Fig. 5, to consider broadly the different aspects of a research problem relating to one or more of the three lower-carbon practices, and where there may be interconnections with other factors.

As well as helping in Step 1 of both pathways, the factor landscape can also be helpful at Step 4 of the becoming interdisciplinary pathway, if a scholar realises their empirical results are not explained by their chosen theory. Examining the factor landscape can assist scholars to identify other factors that may be shaping results. For example, Outcault et al. (2018), wanted to investigate why feedback and commitment strategies hadn’t shifted household cooling practices. Their investigation of their qualitative data revealed *family dynamics* was another key factor shaping this practice. Similarly, a study in Canada of bike-riding found that just *installing separate bike lanes* didn’t switch new people into bike-riding (Mitra et al. 2021). Knowing that other factors, including *social norms* and *habit*, affects whether people engage in bike-riding (Javaid et al. 2020), can help to explain this gap in implementation. Scholars can use the factor landscape to identify other ‘likely suspects’ worth investigating if there are unexpected findings or a gap in implementation.

In the two pathways, both Step 2 (interdisciplinary at the outset) and Step 5 (becoming interdisciplinary) are deciding which disciplines are relevant. For a collaborative inquiry, the decisions made at this stage will determine which scholars are invited to contribute. Many interdisciplinary processes break this down into two stages: an *expansive* stage of considering which disciplines are relevant to the problem, and a *focusing* stage of deciding which disciplines are more central to the problem. When at the focusing stage, an important consideration for scholars is whether they wish to direct their attention to the micro-, meso-, or macro-level. If they are taking a place-based approach, they may choose to consider how all three levels affect people in that place. If they are looking at a particular group of people, then they may wish to focus predominantly at the micro- or meso-level. As outlined in  the “Most used theories” section, theories tend to specialise at one or another level of analysis.

**Fig. 6:**
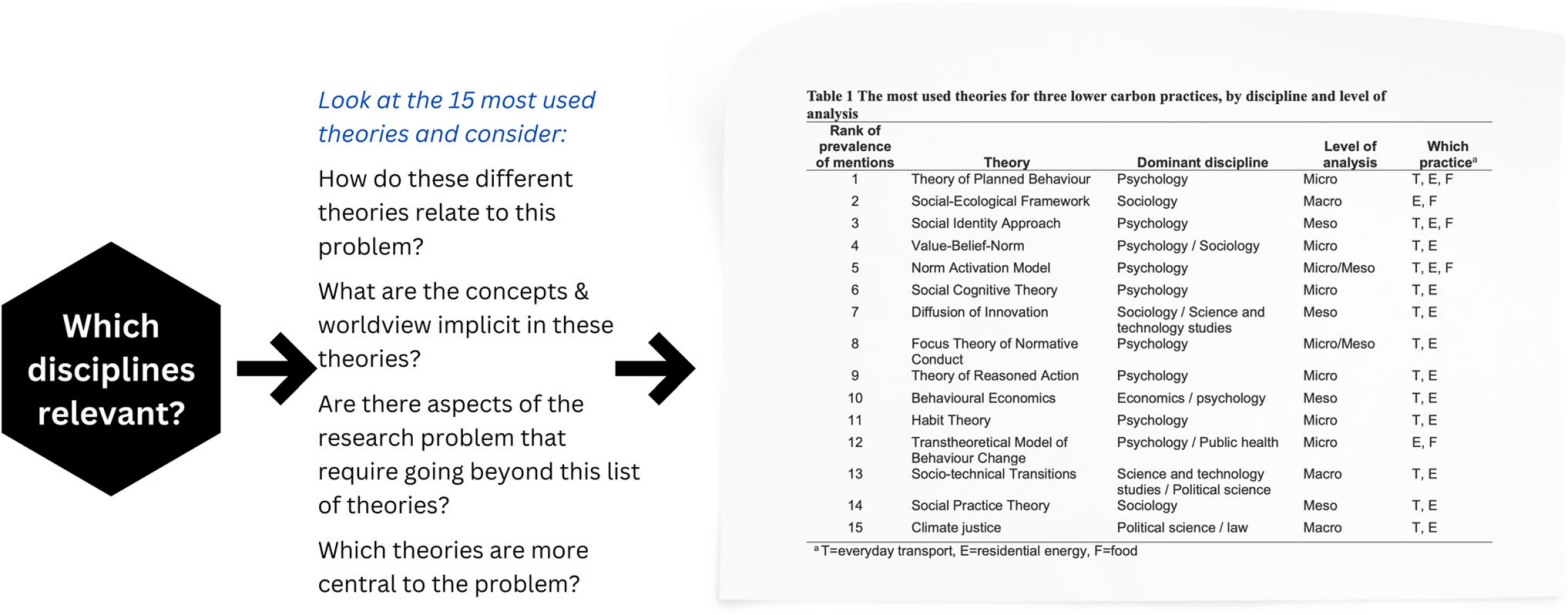
Analysing which disciplines are relevant using the 15 most used theories

The second tool, the most used theories, is helpful at this stage of research. As shown in Fig. 6, Table 2, along with the description of the theories in Table 3, provides a starting point for considering which theories within which disciplines are relevant to a research problem relating to one or more of the three lower-carbon practices. They also assist in considering which theories are useful at which level of analysis, and which theories are more central to the research problem.

The author's (Boddington 2025) inquiry into when householders would consider the adoption of lower-carbon stovetops is an example of an inquiry that was interdisciplinary at the outset. The study was predominantly based on *social practice theory* but was also influenced by *social identity theory*, both meso-level theories. The inquiry used the idea of ‘social meaning’ to bridge theories because both social practice theory and social identity theory consider people’s practices to be shaped by social forces, although they differ in their level of analysis between society and specific social groups. Using a concept to bridge theories is an example of the *connecting* type of interdisciplinary integration discussed in the "Interdisciplinary processes" section and helped to explain what was shaping the actions of different groups of participants. Another theory, *diffusion of innovation* theory, helped to explain participants’ perceptions of induction stovetops, an example of *weaving*, where insights from a discipline are put next to insights from another discipline, to provide a more holistic explanation of social complexity.

The most used theories can also be helpful in disciplinary inquiries that become interdisciplinary at Step 4, by helping scholars identify which theories may assist. Hargreaves’ (2010) inquiry into a workplace’s adoption of green initiatives is an example of this. The inquiry was grounded in social practice theory. Hargreaves found there were power struggles in the workplace between facilities management actors and the actors leading the introduction of the green initiatives. The facilities management group had a more established position and discourses than initiative leaders, leading to the non- or weak- implementation of some of the initiatives. Hargreaves added theories of power, as used by Foucault and others, to argue that the power struggles were a contest over what it meant to be a good employee, an example of the *adding* approach to integration*,* using power theories to supplement social practice theory to more fully explain these results. While this, and the earlier example, are inquiries by individual scholars, there are also examples of collaborative teams using multiple theories to explore lower-carbon practices (Ransan-Cooper et al. 2022; Julsrud and Aasen 2024).

## Discussion

The present study aimed to assist scholars in engaging with the social complexity of three lower-carbon practices by creating two tools and providing a guide to interdisciplinary processes. The first tool, the factor landscape, assists scholars in exploring the different factors of social complexity. It assists with analysing research problems and considering what else might be shaping unexpected empirical results. The second tool, the most used theories, assists scholars in considering what disciplines and theories are relevant to a research problem relating to one or more three lower-carbon practices. They can use this as the basis for inviting scholars to join an interdisciplinary collaboration or as a starting point for themselves developing adequacy in relevant disciplines.

It is worth considering how researchers should engage with the social complexity of lower-carbon practices. Several scholars normatively argue that researchers should not simplify or flatten out the nuances and complexities of this messy reality but instead render these ‘realities’ in their complexity (Castree et al. 2014; Schipper et al. 2021). Schipper et al. (2021) make the case that the task of researchers is to *hold* the different ways that people engage with lower-carbon practices, rather than to try to synthesise them for the purposes of making policy prescriptions. Castree et al. (2014, 763) similarly argue that scholars should normatively aspire to produce “plural representations” of solutions “that are reflective of divergent human values and aspirations”.

In turn this raises the question about the degree to which interventions to enable lower-carbon practices may need to be tailored for different peoples across diverse contexts (Nielsen et al. 2020; Schipper et al. 2021; Fell et al. 2022; Bingley et al. 2023). Sometimes, there will be enough analogous factors that solutions that work in one context, including through engaging peoples’ aspirations, will work in another (Krohn 2017). In the end, though, this can only be determined empirically, with the implication being that there needs to be much more social research to engage with the potential variation in social responses to lower-carbon practices. The present study provides tools for scholars to begin to meet this need.

### Limitations

The present study has some limitations. Firstly, while it identifies most used theories for the exploration of three lower-carbon practices, it does not encompass other ways of knowing outside of the academy, such as indigenous knowledges (Barry and Born 2013; Schipper et al. 2021). In identifying the most used theories to study three lower-carbon practices, it excludes newer theories and approaches, such as relational paradigms (Böhme et al. 2022). Finally, while it introduces some approaches to working with multiple theories, it does not engage with other forms of interdisciplinarity and trans-disciplinarity (Barry and Born 2013; Klein 2017; Pohl et al. 2021). It also introduces, but does not provide a full description of the obstacles to interdisciplinary practice, including established habits, cognitive preferences, and a system that more readily recognises and funds certain forms of research (Shove 2010; Castree et al. 2014; Sulik et al. 2025).

## Conclusion

This study encourages scholars to engage with the social complexities of lower-carbon practices, try interdisciplinary processes, and see their work as part of the process of sharing viewpoints on groups and processes of change. It introduces two analytical tools—the factor landscape and most used theories—with guidance for their application in research. Sustained action is needed to accelerate lower-carbon transitions in high-income countries. These tools provide practical means for exploring pathways suited to the complexity of the world.

## Supplementary Information

Below is the link to the electronic supplementary material.

Supplementary file1 (PDF 483 KB)

## Data Availability

This study does not generate new data.

## References

[CR1] Ajzen, I. 1991. The theory of planned behavior. *Organizational Behavior and Human Decision Processes* 50: 179–211.

[CR2] Albarracín, D., B. Fayaz-Farkhad, and J.A. Granados Samayoa. 2024. Determinants of behaviour and their efficacy as targets of behavioural change interventions. *Nature Reviews Psychology* 3: 377–392. 10.1038/s44159-024-00305-0. 10.1038/s44159-024-00305-0 PMC12407158 40909436

[CR3] Baker, K.J., R. Mould, and S. Restrick. 2018. Rethink fuel poverty as a complex problem. *Nature Energy* 3: 610–612. 10.1038/s41560-018-0204-2.

[CR4] Bamberg, S. 2013. Applying the stage model of self-regulated behavioral change in a car use reduction intervention. *Journal of Environmental Psychology* 33: 68–75. 10.1016/j.jenvp.2012.10.001.

[CR5] Bamberg, S., and M. Schulte. 2018. Processes of change. In *Environmental psychology: An introduction*, 2nd ed., ed. L. Steg and J. I. M. De Groot, 307–318. Hoboken: Wiley. 10.1002/9781119241072.ch30.

[CR6] Bandura, A. 1999. Social cognitive theory: An agentic perspective. *Asian Journal of Social Psychology* 2: 21–41. 10.1111/1467-839X.00024. 10.1146/annurev.psych.52.1.1 11148297

[CR7] Barry, A., and Born. 2013. Interdisciplinarity: Reconfigurations of the social and natural sciences. In *Interdisciplinarity: Reconfigurations of the social and natural sciences*, ed. A. Barry and G. Born, 1–56. London: Taylor and Francis Group. 10.4324/9780203584279.

[CR8] Bartiaux, F., K. Gram-Hanssen, P. Fonseca, L. Ozoliņa, and T.H. Christensen. 2014. A practice–theory approach to homeowners’ energy retrofits in four European areas. *Building Research & Information* 42: 525–538. 10.1080/09613218.2014.900253.

[CR9] Bergquist, M., M. Thiel, M.H. Goldberg, and S. van der Linden. 2023. Field interventions for climate change mitigation behaviors: A second-order meta-analysis. *Proceedings of the National Academy of Sciences* 120: e2214851120. 10.1073/pnas.2214851120. 10.1073/pnas.2214851120 PMC10068847 36943888

[CR10] Bingley, W.J., S.A. Haslam, C. Haslam, M.J. Hornsey, and F. Mols. 2023. Why a group-level analysis is essential for effective public policy: The case for a g-frame. *Behavioral and Brain Sciences* 46: e148. 10.1017/S0140525X23000894. 37646303 10.1017/S0140525X23000894

[CR11] Boddington, S.J. 2025. Cooking up change: Identity, practice, and policy in Australian household stovetop electrification. *Energy Research & Social Science* 119: 103860. 10.1016/j.erss.2024.103860.

[CR12] Böhme, J., Z. Walsh, and C. Wamsler. 2022. Sustainable lifestyles: Towards a relational approach. *Sustainability Science* 17: 2063–2076. 10.1007/s11625-022-01117-y.

[CR13] Böhmelt, T., M. Böker, and H. Ward. 2016. Democratic inclusiveness, climate policy outputs, and climate policy outcomes. *Democratization* 23: 1272–1291. 10.1080/13510347.2015.1094059.

[CR14] Bouman, T., L. Steg, and S.J. Zawadzki. 2020. The value of what others value: When perceived biospheric group values influence individuals’ pro-environmental engagement. *Journal of Environmental Psychology* 71: 101470. 10.1016/j.jenvp.2020.101470.

[CR15] Castree, N., W.M. Adams, J. Barry, D. Brockington, B. Büscher, E. Corbera, D. Demeritt, R. Duffy, et al. 2014. Changing the intellectual climate. *Nature Climate Change* 4: 763–768. 10.1038/nclimate2339.

[CR16] Centola, D. 2021. *Change: How to make big things happen*, 1st ed. New York: Little, Brown Spark.

[CR17] Centola, D., J. Becker, D. Brackbill, and A. Baronchelli. 2018. Experimental evidence for tipping points in social convention. *Science* 360: 1116–1119. 10.1126/science.aas8827. 29880688 10.1126/science.aas8827

[CR18] Chancel, L. 2022. Global carbon inequality over 1990–2019. *Nature Sustainability* 5: 931–938. 10.1038/s41893-022-00955-z.

[CR19] Chatzigeorgiou, I.M., and G.T. Andreou. 2021. A systematic review on feedback research for residential energy behavior change through mobile and web interfaces. *Renewable Sustainable Energy Review*. 10.1016/j.rser.2020.110187.

[CR20] Christidis, R., M. Lock, T. Walker, M. Egan, and J. Browne. 2021. Concerns and priorities of Aboriginal and Torres Strait Islander peoples regarding food and nutrition: A systematic review of qualitative evidence. *International Journal for Equity in Health*. 10.1186/s12939-021-01551-x. 10.1186/s12939-021-01551-x PMC8499519 34620180

[CR21] Cialdini, R.B., and N.J. Goldstein. 2004. Social influence: Compliance and conformity. *Annual Review of Psychology* 55: 591–621. 10.1146/annurev.psych.55.090902.142015. 10.1146/annurev.psych.55.090902.142015 14744228

[CR22] Cialdini, R.B., C.A. Kallgren, and R.R. Reno. 1991. A Focus Theory of Normative Conduct: A Theoretical Refinement and Reevaluation of the Role of Norms in Human Behavior. In *Advances in Experimental Social Psychology*, Vol. 24, 201–234. Amsterdam: Elsevier. 10.1016/S0065-2601(08)60330-5.

[CR23] Corradi, C., E. Sica, and P. Morone. 2023. What drives electric vehicle adoption? Insights from a systematic review on European transport actors and behaviours. *Energy Research & Social Science*. 10.1016/j.erss.2022.102908.

[CR24] Daramy-Williams, E., J. Anable, and S. Grant-Muller. 2019. A systematic review of the evidence on plug-in electric vehicle user experience. *Transportation Research Part D: Transport and Environment* 71: 22–36. 10.1016/j.trd.2019.01.008.

[CR25] Darnton, A., and J. Horne. 2013. *Influencing behaviours: Moving beyond the individual: A user guide to the ISM tool*. Edinburgh: Scottish Government.

[CR26] De Dominicis, S., R. Sokoloski, C.M. Jaeger, and P.W. Schultz. 2019. Making the smart meter social promotes long-term energy conservation. *Palgrave Communications* 5: 51. 10.1057/s41599-019-0254-5.

[CR27] De Groot, J.I.M., and L. Steg. 2009. Morality and prosocial behavior: The role of awareness, responsibility, and norms in the Norm Activation Model. *The Journal of Social Psychology* 149: 425–449. 10.3200/SOCP.149.4.425-449. 19702104 10.3200/SOCP.149.4.425-449

[CR28] Dearing, J.W. 2009. Applying diffusion of innovation theory to intervention development. *Research on Social Work Practice* 19: 503–518. 10.1177/1049731509335569. 20976022 10.1177/1049731509335569 PMC2957672

[CR29] Dueñas-Ocampo, S., W. Eichhorst, and P. Newton. 2023. Plant-based and cultivated meat in the United States: A review and research agenda through the lens of socio-technical transitions. *Journal of Cleaner Production* 405: 136999. 10.1016/j.jclepro.2023.136999.

[CR30] Dwarkasing, C. 2023. Inequality determined social outcomes of low-carbon transition policies: A conceptual meta-review of justice impacts. *Energy Research & Social Science*. 10.1016/j.erss.2023.102974.

[CR31] Fell, M.J., K. Roelich, and L. Middlemiss. 2022. Realist approaches in energy research to support faster and fairer climate action. *Nature Energy* 7: 916–922. 10.1038/s41560-022-01093-8.

[CR32] Fielding, K.S., and M.J. Hornsey. 2016. A social identity analysis of climate change and environmental attitudes and behaviors: Insights and opportunities. *Frontiers in Psychology*. 10.3389/fpsyg.2016.00121. 10.3389/fpsyg.2016.00121 PMC4749709 26903924

[CR33] Fishbein, M., and I. Ajzen. 2009. *Predicting and Changing Behavior: The Reasoned Action Approach*. Oxford, UK: Taylor & Francis Group.

[CR34] Foster, C., P. Kelly, H.A.B. Reid, N. Roberts, E.M. Murtagh, D.K. Humphreys, J. Panter, and K. Milton. 2018. What works to promote walking at the population level? A systematic review. *British Journal of Sports Medicine* 52: 807–812. 10.1136/bjsports-2017-098953. 29858468 10.1136/bjsports-2017-098953 PMC6258897

[CR35] Friman, M., J. Huck, and L. Olsson. 2017. Transtheoretical model of change during travel behavior interventions: An integrative review. *International Journal of Environmental Research and Public Health* 14: 581. 10.3390/ijerph14060581. 28556810 10.3390/ijerph14060581 PMC5486267

[CR36] Friman, M., R. Maier, and L.E. Olsson. 2019. Applying a motivational stage-based approach in order to study a temporary free public transport intervention. *Transport Policy* 81: 173–183. 10.1016/j.tranpol.2019.06.012.

[CR37] Geels, F.W., F. Berkhout, and D.P. Van Vuuren. 2016. Bridging analytical approaches for low-carbon transitions. *Nature Climate Change* 6: 576–583. 10.1038/nclimate2980.

[CR38] Geels, F.W., B.K. Sovacool, T. Schwanen, and S. Sorrell. 2017a. Sociotechnical transitions for deep decarbonization. *Science* 357: 1242–1244. 10.1126/science.aao3760. 28935795 10.1126/science.aao3760

[CR39] Geels, F.W., B.K. Sovacool, T. Schwanen, and S. Sorrell. 2017b. The socio-technical dynamics of low-carbon transitions. *Joule* 1: 463–479. 10.1016/j.joule.2017.09.018.

[CR40] Grant, M.J., and A. Booth. 2009. A typology of reviews: An analysis of 14 review types and associated methodologies: A typology of reviews. *Health Information & Libraries Journal* 26: 91–108. 10.1111/j.1471-1842.2009.00848.x. 19490148 10.1111/j.1471-1842.2009.00848.x

[CR41] Graziano, M., and K. Gillingham. 2015. Spatial patterns of solar photovoltaic system adoption: The influence of neighbors and the built environment. *Journal of Economic Geography* 15: 815–839. 10.1093/jeg/lbu036.

[CR42] Gulliver, R.E., A. Vachette, and S. Boddington. 2023. How Australian environmental non-governmental organisations frame and enact climate justice. *npj Climate Action* 2: 18. 10.1038/s44168-023-00049-2.

[CR43] Hackmann, H., S.C. Moser, A.L. Hackman, and L.S. Clair. 2014. The social heart of global environmental change. *Nature Climate Change* 4: 653–655. 10.1038/nclimate2320.

[CR44] Hampton, S., and R. Adams. 2018. Behavioural economics vs social practice theory: Perspectives from inside the United Kingdom government. *Energy Research & Social Science* 46: 214–224. 10.1016/j.erss.2018.07.023.

[CR45] Hanke, F., K. Grossmann, and L. Sandmann. 2023. Excluded despite their support—The perspectives of energy-poor households on their participation in the German energy transition narrative. *Energy Research & Social Science* 104: 103259. 10.1016/j.erss.2023.103259.

[CR46] Hargreaves, T. 2010. Making pro-environmental behaviour work: An ethnographic case study of practice, process and power in the workplace. Doctoral dissertation, University of East Anglia.

[CR47] Hazard, L., M. Cerf, C. Lamine, D. Magda, and P. Steyaert. 2019. A tool for reflecting on research stances to support sustainability transitions. *Nature Sustainability* 3: 89–95. 10.1038/s41893-019-0440-x.

[CR48] Hoffmann, C., C. Abraham, M.P. White, S. Ball, and S.M. Skippon. 2017. What cognitive mechanisms predict travel mode choice? A systematic review with meta-analysis. *Transport Reviews* 37: 631–652. 10.1080/01441647.2017.1285819.

[CR49] Hofmann, B., U. Reber, P. Ammann, J. Dötzer, J. Mark, C. McCallum, M. Wiget, and L. Zachmann. 2025. A typology of interdisciplinary collaborations: Insights from agri-food transformation research. *Sustainability Science* 20: 1791–1808. 10.1007/s11625-025-01702-x. 40922901 10.1007/s11625-025-01702-x PMC12414061

[CR50] Hoolohan, C., C. McLachlan, and S. Mander. 2018. Food related routines and energy policy: A focus group study examining potential for change in the United Kingdom. *Energy Research & Social Science* 39: 93–102. 10.1016/j.erss.2017.10.050.

[CR51] Horvat, A., V. Fogliano, and P.A. Luning. 2020. Modifying the Bass diffusion model to study adoption of radical new foods–The case of edible insects in the Netherlands. *Edited by Jarosław Jankowski. PLOS ONE* 15: e0234538. 10.1371/journal.pone.0234538. 10.1371/journal.pone.0234538 PMC7289433 32525950

[CR52] Hu, S., D. Yan, E. Azar, and F. Guo. 2020. A systematic review of occupant behavior in building energy policy. *Building and Environment.*10.1016/j.buildenv.2020.106807.

[CR53] Ivanova, D., K. Stadler, K. Steen-Olsen, R. Wood, G. Vita, A. Tukker, and E.G. Hertwich. 2016. Environmental impact assessment of household consumption: Environmental impact assessment of household consumption. *Journal of Industrial Ecology* 20: 526–536. 10.1111/jiec.12371.

[CR54] Ivanova, D., J. Barrett, D. Wiedenhofer, B. Macura, M. Callaghan, and F. Creutzig. 2020. Quantifying the potential for climate change mitigation of consumption options. *Environmental Research Letters* 15: 093001. 10.1088/1748-9326/ab8589.

[CR55] Javaid, A., F. Creutzig, and S. Bamberg. 2020. Determinants of low-carbon transport mode adoption: Systematic review of reviews. *Environmental Research Letters* 15: 103002. 10.1088/1748-9326/aba032.

[CR56] Julsrud, T.E., and M. Aasen. 2024. “Robots taking over the world… fantastic!” Understanding social representations, familiarity and visions of experiments with autonomous public transportation. *Energy Research & Social Science* 115: 103646. 10.1016/j.erss.2024.103646.

[CR57] Kahneman, D. 2011. *Thinking, fast and slow*, 1st ed. New York: Farrar, Straus and Giroux.

[CR58] Kamali Saraji, M., and D. Streimikiene. 2023. Challenges to the low carbon energy transition: A systematic literature review and research agenda. *Energy Strategy Reviews* 49: 101163. 10.1016/j.esr.2023.101163.

[CR59] Kastner, I., and P.C. Stern. 2015. Examining the decision-making processes behind household energy investments: A review. *Energy Research & Social Science* 10: 72–89. 10.1016/j.erss.2015.07.008.

[CR60] Keesing, R.M. 1974. Theories of culture. *Annual Review of Anthropology* 3: 73–97. 10.1146/annurev.an.03.100174.000445.

[CR61] Kellermeyer, L., B. Harnke, and S. Knight. 2018. Covidence and Rayyan. *Journal of the Medical Library Association*. 10.5195/jmla.2018.513.

[CR62] Klein, J.T. 2017. 3 Typologies of interdisciplinarity: The boundary work of definition. In *The Oxford handbook of interdisciplinarity*, 2nd ed., ed. R. Frodeman. Oxford, England: Oxford University Press.

[CR63] Klöckner, C.A., and E. Matthies. 2004. How habits interfere with norm-directed behaviour: A normative decision-making model for travel mode choice. *Journal of Environmental Psychology* 24: 319–327. 10.1016/j.jenvp.2004.08.004.

[CR64] Koide, R., M. Lettenmeier, L. Akenji, V. Toivio, A. Amellina, A. Khodke, A. Watabe, and S. Kojima. 2021. Lifestyle carbon footprints and changes in lifestyles to limit global warming to 1.5 °C, and ways forward for related research. *Sustainability Science* 16: 2087–2099. 10.1007/s11625-021-01018-6.

[CR65] Krohn, W. 2017. 4 Interdisciplinary cases and disciplinary knowledge: Epistemic challenges of interdisciplinary research. In *The Oxford handbook of interdisciplinarity*, 2nd ed., ed. R. Frodeman. Oxford, England: Oxford University Press.

[CR66] Kurz, T., A.M.B. Prosser, A. Rabinovich, and S. O’Neill. 2020. Could vegans and lycra cyclists be bad for the planet? Theorizing the role of moralized minority practice identities in processes of societal‐level change. *Journal of Social Issues* 76: 86–100. 10.1111/josi.12366.

[CR67] Lang, M., R. Lane, K. Zhao, S. Tham, K. Woolfe, and R. Raven. 2021. Systematic review: Landlords’ willingness to retrofit energy efficiency improvements. *Journal of Cleaner Production* 303: 127041. 10.1016/j.jclepro.2021.127041.

[CR68] Lanzini, P., and S.A. Khan. 2017. Shedding light on the psychological and behavioral determinants of travel mode choice: A meta-analysis. *Transportation Research Part F: Traffic Psychology and Behaviour* 48: 13–27. 10.1016/j.trf.2017.04.020.

[CR69] Lind, H.B., T. Nordfjærn, S. H. Jørgensen, and T. Rundmo. 2015. The value-belief-norm theory, personal norms and sustainable travel mode choice in urban areas. *Journal of Environmental Psychology* 44: 119–125. 10.1016/j.jenvp.2015.06.001.

[CR70] Lindsay, S., and D.-L. Lamptey. 2019. Pedestrian navigation and public transit training interventions for youth with disabilities: A systematic review. *Disability and Rehabilitation* 41: 2607–2621. 10.1080/09638288.2018.1471165. 29741968 10.1080/09638288.2018.1471165

[CR71] Lindvall, D., and M. Karlsson. 2024. Exploring the democracy-climate nexus: A review of correlations between democracy and climate policy performance. *Climate Policy* 24: 87–103. 10.1080/14693062.2023.2256697.

[CR72] Martiskainen, M., B.K. Sovacool, M. Lacey-Barnacle, D. Hopkins, K.E.H. Jenkins, N. Simcock, G. Mattioli, and S. Bouzarovski. 2021. New dimensions of vulnerability to energy and transport poverty. *Joule* 5: 3–7. 10.1016/j.joule.2020.11.016.

[CR73] Marzi, I., Y. Demetriou, and A.K. Reimers. 2018. Social and physical environmental correlates of independent mobility in children: A systematic review taking sex/gender differences into account. *International Journal of Health Geographics*. 10.1186/s12942-018-0145-9. 10.1186/s12942-018-0145-9 PMC6029402 29970117

[CR74] Meadowcroft, J., and D. Rosenbloom. 2023. Governing the net-zero transition: Strategy, policy, and politics. *Proceedings of the National Academy of Sciences* 120: e2207727120. 10.1073/pnas.2207727120. 10.1073/pnas.2207727120 PMC10666100 37956296

[CR75] Menken, S., and M. Keestra. 2016. *An introduction to interdisciplinary research: Theory and practice*. Amsterdam: Amsterdam University Press.

[CR76] Miller, D.T., and D.A. Prentice. 2016. Changing norms to change behavior. *Annual Review of Psychology* 67: 339–361. 10.1146/annurev-psych-010814-015013. 10.1146/annurev-psych-010814-015013 26253542

[CR77] Mitra, R., A. Khachatryan, and P.M. Hess. 2021. Do new urban and suburban cycling facilities encourage more bicycling? *Transportation Research Part d: Transport and Environment* 97: 102915. 10.1016/j.trd.2021.102915.

[CR78] Moon, K., C. Cvitanovic, D.A. Blackman, I.R. Scales, and N.K. Browne. 2021. Five questions to understand epistemology and its influence on integrative marine research. *Frontiers in Marine Science* 8: 574158. 10.3389/fmars.2021.574158.

[CR79] Mylan, J., C. Morris, E. Beech, and F.W. Geels. 2019. Rage against the regime: Niche-regime interactions in the societal embedding of plant-based milk. *Environmental Innovation and Societal Transitions* 31: 233–247. 10.1016/j.eist.2018.11.001.

[CR80] Nash, N., L. Whitmarsh, S. Capstick, T. Hargreaves, W. Poortinga, G. Thomas, E. Sautkina, and D. Xenias. 2017. Climate-relevant behavioral spillover and the potential contribution of social practice theory. *WIREs Climate Change*. 10.1002/wcc.481.

[CR81] Newell, P. 2022. Climate justice. *The Journal of Peasant Studies* 49: 915–923. 10.1080/03066150.2022.2080062.

[CR82] Newell, P., S. Srivastava, L.O. Naess, G.A. Torres Contreras, and R. Price. 2021. Toward transformative climate justice: An emerging research agenda. *WIREs Climate Change*. 10.1002/wcc.733.

[CR83] Newell, W.H. 2006. Decision making in interdisciplinary studies. In *Handbook of decision making*, ed. G. Morcol. Oxford, UK: Taylor & Francis Group.

[CR84] Nielsen, K.S., P.C. Stern, T. Dietz, J.M. Gilligan, D.P. Van Vuuren, M.J. Figueroa, C. Folke, W. Gwozdz, et al. 2020. Improving climate change mitigation analysis: A framework for examining feasibility. *One Earth* 3: 325–336. 10.1016/j.oneear.2020.08.007.

[CR85] Olsson, L.E., R. Maier, and M. Friman. 2019. Why do they ride with others? Meta-analysis of factors influencing travelers to carpool. *Sustainability.*10.3390/su11082414.

[CR86] Orlove, B., R. Shwom, E. Markowitz, and S.-M. Cheong. 2020. Climate decision-making. *Annual Review of Environment and Resources* 45: 271–303. 10.1146/annurev-environ-012320-085130.

[CR87] Outcault, S., A. Sanguinetti, and M. Pritoni. 2018. Using social dynamics to explain uptake in energy saving measures: Lessons from space conditioning interventions in Japan and California. *Energy Research & Social Science* 45: 276–286. 10.1016/j.erss.2018.07.017.

[CR88] Patterson, J.J. 2022. Culture and identity in climate policy. *WIREs Climate Change*. 10.1002/wcc.765.

[CR89] Pearson, A.R., J.P. Schuldt, and R. Romero-Canyas. 2016. Social climate science: A new vista for psychological science. *Perspectives on Psychological Science* 11: 632–650. 10.1177/1745691616639726. 27694459 10.1177/1745691616639726

[CR90] Piatkowski, D., and M. Bopp. 2021. Increasing bicycling for transportation: A systematic review of the literature. *Journal of Urban Planning and Development*. 10.1061/(ASCE)UP.1943-5444.0000693.

[CR91] Pohl, C., J.T. Klein, S. Hoffmann, C. Mitchell, and D. Fam. 2021. Conceptualising transdisciplinary integration as a multidimensional interactive process. *Environmental Science & Policy* 118: 18–26. 10.1016/j.envsci.2020.12.005.

[CR92] Pollard, T.M., and J.M. Wagnild. 2017. Gender differences in walking (for leisure, transport and in total) across adult life: A systematic review. *BMC Public Health*. 10.1186/s12889-017-4253-4. 10.1186/s12889-017-4253-4 PMC5397769 28427376

[CR93] Ransan-Cooper, H., M. Shaw, B.C.P. Sturmberg, and L. Blackhall. 2022. Neighbourhood batteries in Australia: Anticipating questions of value conflict and (in)justice. *Energy Research & Social Science* 90: 102572. 10.1016/j.erss.2022.102572.

[CR94] Ravi, K., N. Fields, and H. Dabelko-Schoeny. 2021. Outdoor spaces and buildings, transportation, and environmental justice: A qualitative interpretive meta-synthesis of two age-friendly domains. *Journal of Transport & Health*. 10.1016/j.jth.2020.100977.

[CR95] Repko, A., W. Newell, and R. Szostak. 2012. The Interdisciplinary Research Process. In *Case studies in interdisciplinary research*. California: SAGE Publications, Inc. 10.4135/9781483349541.

[CR96] Repko, A.F. 2006. Disciplining interdisciplinarity: The case for textbooks. *Issues in Integrative Studies* 24: 112–142.

[CR97] Riazi, N., K. Wunderlich, L. Yun, D. Paterson, and G. Faulkner. 2022. Social-ecological correlates of children’s independent mobility: A systematic review. *International Journal of Environmental Research and Public Health*. 10.3390/ijerph19031604. 10.3390/ijerph19031604 PMC8835222 35162626

[CR98] Roberts, C., F.W. Geels, M. Lockwood, P. Newell, H. Schmitz, B. Turnheim, and A. Jordan. 2018. The politics of accelerating low-carbon transitions: Towards a new research agenda. *Energy Research & Social Science* 44: 304–311. 10.1016/j.erss.2018.06.001.

[CR99] Rogers, E.M. 1995. Chapter 6 Attributes of Innovations and their rate of adoption. In *Diffusion of innovations*. New York: The Free Press.

[CR100] Rothman, L., A.K. Macpherson, T. Ross, and R.N. Buliung. 2018. The decline in active school transportation (AST): A systematic review of the factors related to AST and changes in school transport over time in North America. *Preventive Medicine* 111: 314–322. 10.1016/j.ypmed.2017.11.018. 29155222 10.1016/j.ypmed.2017.11.018

[CR101] Sallis, J.F., R.B. Cervero, W. Ascher, K.A. Henderson, M.K. Kraft, and J. Kerr. 2006. An ecological approach to creating active living communities. *Annual Review of Public Health* 27: 297–322. 10.1146/annurev.publhealth.27.021405.102100. 10.1146/annurev.publhealth.27.021405.102100 16533119

[CR102] Scepanovic, S., M. Warnier, and J. Nurminen. 2017. The role of context in residential energy interventions: A meta review. *Renewable and Sustainable Energy Reviews.* 77: 1146–1168. 10.1016/j.rser.2019.04.033.

[CR103] Scheller, F. 2024. Green or greedy: The relationship between perceived benefits and homeowners’ intention to adopt residential low-carbon technologies. *Energy Research and Social Science* 108: 103388. 10.1016/j.erss.2023.103388.

[CR104] Schipper, E.L.F., N.K. Dubash, and Y. Mulugetta. 2021. Climate change research and the search for solutions: Rethinking interdisciplinarity. *Climatic Change* 168: 18. 10.1007/s10584-021-03237-3. 34690385 10.1007/s10584-021-03237-3 PMC8520790

[CR105] Schwartz, S.H. 1977. Normative Influences on Altruism. In *Advances in experimental social psychology*, Vol. 10, 221–279. Amsterdam: Elsevier. 10.1016/S0065-2601(08)60358-5.

[CR106] Shove, E. 2010. Beyond the ABC: Climate change policy and theories of social change. *Environment and Planning a: Economy and Space* 42: 1273–1285. 10.1068/a42282.

[CR107] Shove, E., M. Pantzar, and M. Watson. 2012. *The dynamics of social practice: Everyday life and how it changes*. London: Sage.

[CR108] Soininen, N., J.B. Ruhl, B.A. Cosens, and L. Gunderson. 2025. Governing complexity: A comparative assessment of four governance models with applications to climate change mitigation and adaptation. *Environmental Innovation and Societal Transitions* 57: 101020. 10.1016/j.eist.2025.101020.

[CR109] Southerton, D. 2007. Time pressure, technology and gender: The conditioning of temporal experiences in the UK. Edited by S Jacqueline. *Equal Opportunities International* 26: 113–128. 10.1108/02610150710732195.

[CR110] Sovacool, B.K., and M.-C. Brisbois. 2019. Elite power in low-carbon transitions: A critical and interdisciplinary review. *Energy Research & Social Science* 57: 101242. 10.1016/j.erss.2019.101242.

[CR111] Sovacool, B.K., and D.J. Hess. 2017. Ordering theories: Typologies and conceptual frameworks for sociotechnical change. *Social Studies of Science* 47: 703–750. 10.1177/0306312717709363. 28641502 10.1177/0306312717709363 PMC5648049

[CR112] Sovacool, B.K., P. Newell, S. Carley, and J. Fanzo. 2022. Equity, technological innovation and sustainable behaviour in a low-carbon future. *Nature Human Behaviour* 6: 326–337. 10.1038/s41562-021-01257-8. 10.1038/s41562-021-01257-8 35102347

[CR113] Spears, R. 2021. Social influence and group identity. *Annual Review of Psychology* 72: 367–390. 10.1146/annurev-psych-070620-111818. 10.1146/annurev-psych-070620-111818 32931718

[CR114] Steinbach, R., J. Green, J. Datta, and P. Edwards. 2011. Cycling and the city: A case study of how gendered, ethnic and class identities can shape healthy transport choices. *Social Science & Medicine* 72: 1123–1130. 10.1016/j.socscimed.2011.01.033. 21396761 10.1016/j.socscimed.2011.01.033

[CR115] Stern, P.C. 2000. New environmental theories: Toward a coherent theory of environmentally significant behavior. *Journal of Social Issues* 56: 407–424. 10.1111/0022-4537.00175.

[CR116] Stern, P.C., T. Dietz, T. Abel, G.A. Guagnano, and L. Kalof. 1999. A value-belief-norm theory of support for social movements: The case of environmentalism. *Human Ecology Review* 1: 81–97.

[CR117] Sulik, J., N. Rim, E. Pontikes, J. Evans, and G. Lupyan. 2025. Differences in psychologists’ cognitive traits are associated with scientific divides. *Nature Human Behaviour* 9: 1147–1161. 10.1038/s41562-025-02153-1. 10.1038/s41562-025-02153-1 PMC12185325 40246997

[CR118] Tajfel, H. 1981. *Human groups and social categories: Studies in social psychology*, vol. 255. Cambridge: Cambridge University Press.

[CR119] Taufik, D., M.C.D. Verain, E.P. Bouwman, and M.J. Reinders. 2019. Determinants of real-life behavioural interventions to stimulate more plant-based and less animal-based diets: A systematic review. *Trends in Food Science & Technology* 93: 281–303. 10.1016/j.tifs.2019.09.019.

[CR120] Tidemann, C., N. Engerer, F. Markham, B. Doran, and J.C.V. Pezzey. 2019. Spatial disaggregation clarifies the inequity in distributional outcomes of household solar PV installation. *Journal of Renewable and Sustainable Energy* 11: 035901. 10.1063/1.5097424.

[CR121] Turner, J.C., M.A. Hogg, P.J. Oakes, S.D. Reicher, and M.S. Wetherell. 1987. *Rediscovering the social group: A self-categorization theory*. Oxford: Basil Blackwell.

[CR122] Turner, J.C., K.J. Reynolds, S.A. Haslam, and K.E. Veenstra. 2006. Reconceptualizing personality: Producing individuality by defining the personal self. In *Individuality and the group: Advances in social identity*, ed. T. Postmes and J. Jetten, 12–36. London, UK: SAGE Publications Ltd. 10.4135/9781446211946.n2.

[CR123] United Nations Environment Programme. 2020. *The emissions gap report 2020*. Nairobi.

[CR124] Valli, C., M. Maraj, A. Prokop-Dorner, C. Kaloteraki, C. Steiner, M. Rabassa, I. Sola, J. Zajac, et al. 2023. People’s values and preferences about meat consumption in view of the potential environmental impacts of meat: A mixed-methods systematic review. *International Journal of Environmental Research and Public Health*. 10.3390/ijerph20010286. 10.3390/ijerph20010286 PMC9819158 36612609

[CR125] Van Der Werff, E., and L. Steg. 2015. One model to predict them all: Predicting energy behaviours with the norm activation model. *Energy Research & Social Science* 6: 8–14. 10.1016/j.erss.2014.11.002.

[CR126] Van Rijnsoever, F.J., and J. Leendertse. 2020. A practical tool for analyzing socio-technical transitions. *Environmental Innovation and Societal Transitions* 37: 225–237. 10.1016/j.eist.2020.08.004.

[CR127] Van Valkengoed, A.M., W. Abrahamse, and L. Steg. 2022. To select effective interventions for pro-environmental behaviour change, we need to consider determinants of behaviour. *Nature Human Behaviour* 6: 1482–1492. 10.1038/s41562-022-01473-w. 10.1038/s41562-022-01473-w 36385176

[CR128] Vesely, S., T. Masson, P. Chokrai, A. M. Becker, I. Fritsche, C.A. Klöckner, L. Tiberio, G. Carrus, et al. 2021. Climate change action as a project of identity: Eight meta-analyses. *Global Environmental Change* 70: 102322. 10.1016/j.gloenvcha.2021.102322.

[CR129] Watson, M., A. Browne, D. Evans, M. Foden, C. Hoolohan, and L. Sharp. 2020. Challenges and opportunities for re-framing resource use policy with practice theories: The change points approach. *Global Environmental Change* 62: 102072. 10.1016/j.gloenvcha.2020.102072.

