# Supplementary material for: Navigating complexity: A guide and tools for interdisciplinary research on lower-carbon practices
Source: Ambio. 2026 Mar 17;55(8):1750–67. doi: 10.1007/s13280-026-02369-z (PMC13319558; doi:10.1007/s13280-026-02369-z)
Supplement: Supplementary file 1 — Supplementary file1 (PDF 483 KB) [file 13280_2026_2369_MOESM1_ESM.pdf]

1. SUPPLEMENTARY MATERIALS

This Supplementary Information has not been peer reviewed.

Article: **Navigating Complexity: A Guide and Tools for Interdisciplinary Research on Lower Carbon Practices**

Journal: **Ambio**

Author: **Sarah J Boddington,**

The Australian National University

sarah.boddington@anu.edu.au

These Supplementary Materials provide additional information on the systematic approach used to identify factors and theories relevant to the shift to lower carbon practices. Section 1 sets out the systematic approach, Section 2 the method for identifying factors, and Section 3 the method for identifying most used theories.

1. SYSTEMATIC APPROACH

The author undertook a systematic search of recent systematic and meta reviews of everyday transport, residential energy, food, and flying practices. This followed the 'realist review' approach outlined by Berrang-Ford and colleagues (2015), which has guided multiple studies (Sherman et al. 2016; Siders 2019; Tenzing 2020). The search was undertaken in Web of Science and Scopus in August 2023. These articles were screened using the criteria listed in Table S1.

Table S1: Criteria for inclusion and exclusion

Inclusion	Exclusion
High income liberal democratic countries	Reviews focused solely on low or middle income countries<br>Reviews focused solely on non-democratic countries
An increase in the population doing one of the selected lower carbon practices or reducing a high carbon practice<br>Focused on the highest impact low carbon practices of everyday transport, residential energy, flights, and food	Health or safety benefits of a low carbon practice<br>The carbon emissions of a low carbon practice<br>Focused on increasing physical activity rather than low carbon transport
Meta review, systematic review, meta-analysis or meta synthesis<br>Published between 2016 and July 2023	Single study articles<br><br>Published earlier than 2016<br>Published after July 2023<br>Not peer-reviewed<br>Not published in English

2. IDENTIFYING FACTORS

Coding of **factors** followed a two-stage inductive process. First, a random sample of 15 reviews from across the three areas of practice inductively yielded 19 important factors:

- Infrastructure/built environment
- New technologies
- Services e.g. public transport, food outlets, tradespeople, shops
- Knowledge of low carbon practice

- Skills
- Social norms including gender
- Narratives
- Associated with life event
- Social networks
- Subjective norms
- Routines and habits
- Sensory factors e.g. neophobia
- Behavioural control / self-efficacy
- Environmental motivation
- Other motivation, e.g. health, saving money
- Knowledge of climate/ environment impact
- Politics and interests
- Cost / financial assistance
- Non-financial incentives or policies

These were used to create an extraction form in Covidence (Kellermeyer et al. 2018; McKeown and Mir 2021), an online program which uploads references from systematic searches, identifies and removes duplicates, and arranges articles for manual review and extraction of relevant information.

Any additional factors were captured under “Other”. Next, these “Other” factors were coded. If an “Other” factor was, in fact, a dimension of one of the original 19 factors, it was coded to that factor, otherwise it was listed as a new factor. This resulted in 6 additional factors:

- Culture
- Family dynamics
- Ease of the practice
- Social support
- Safety
- Emotional responses

Table S2: Factors shaping lower carbon practices*

Factor	Total	Everyday transport (n=60)	Residential energy (n=11)	Food (n=12)	Multi-practice studies that include everyday transport, residential energy or food (n=18)
Cost / financial assistance	29	13	7	4	5
Non-financial incentives or policies	42	27	8	1	6
Safety	22	22	0	0	0
Subjective norms	14	5	2	2	5
Environmental motivation	26	9	4	6	7
Knowledge of environment / climate impact	13	3	4	5	1
Other motivations	40	20	5	8	7
Social norms incl gender	35	18	4	4	9
Narratives	3	1	2	0	0
Associated with life event	5	4	0	0	1
Social networks	11	7	1	1	2
Culture	8	2	0	5	1
Family dynamics	1	0	0	1	0
Social support	11	9	0	2	0
Sensory factors e.g. neophobia	4	0	0	3	1
Behavioural control / self-efficacy	33	18	7	6	2
Emotional responses	3	2	0	1	0
Politics and interests	13	8	1	1	3
Infrastructure / built environment	53	44	5	2	2
New technologies	19	10	5	1	3
Services	31	21	4	4	2
Knowledge of low carbon practice	29	12	7	6	4
Skills	15	10	1	2	2
Ease of the practice	12	6	2	1	3
Routines and habits	17	9	2	2	4

*Population and geography context-specific aspects were *not* coded as factors – for example, education, income, assets, ethnicity, age and weather.

3. IDENTIFYING THEORIES

The author modified the extraction form in Covidence to identify the theories mentioned in the review papers. This included every theory, model or framework that was mentioned in the 101 extracted reviews. There were 151 theories total, with 45 mentioned more than once, and 15 theories mentioned at least four times in at least two out of three domains, as set out in Table S3.

Table S3: Theories mentioned in the review papers

	Theory	Total	Everyday transport (n=60)	Residential energy (n=11)	Food (n=12)	Multi-practice studies (n=18)	Mentioned in at least 2 out of 3 domains?
1	Theory of planned behaviour	27	7	11	2	7	Y
2	Social- ecological framework	20	0	18	2	0	Y
3	Social identity approach	14	2	3	3	6	Y
4	Value-Belief-Norm	10	4	2	0	4	Y
5	Norm Activation Model	9	2	4	1	2	Y
6	Social cognitive theory	9	2	5	0	2	Y
7	Diffusion of innovation	7	3	3	0	1	Y
8	Focus theory of normative conduct	7	2	2	0	3	Y
9	Theory of reasoned action	7	2	4	0	1	Y
10	Behavioural Economics	6	2	4	0	0	Y
11	Habit theory	6	1	4	0	1	Y
12	Transtheoretical model of behaviour change	6	0	4	1	1	Y
13	Socio-technical transitions	5	2	1	0	2	Y
14	Social practice theory	4	2	2	0	0	Y
15	Climate justice	4	1	2	0	1	Y
16	Technology acceptance model	3	1	1	0	1	Y
17	Utility theory	3	1	2	0	0	Y
18	Control theory	2	1	1	0	0	Y
19	Social networks	6	0	4	0	2	
20	Construal level theory	5	0	0	0	5	
21	Sensory sciences	4	0	0	4	0	
22	Theory of Interpersonal Behavior	4	0	3	0	1	
23	motivation theory	3	0	0	1	2	
24	Political economy (incl discourse analysis)	3	0	3	0	0	
25	Self-determination theory	3	0	2	0	1	
26	Self-perception theory	3	0	0	0	3	

	Theory	Total	Everyday transport (n=60)	Residential energy (n=11)	Food (n=12)	Multi-practice studies (n=18)	Mentioned in at least 2 out of 3 domains?
27	Social comparison	3	0	2	0	1	
28	Transition studies	3	0	2	0	1	
29	ABC theory	2	1	0	0	1	
30	Active Living by Design Community Action Model	2	0	2	0	0	
31	Attitude Theory	2	0	1	0	1	
32	Cognitive dissonance theory	2	0	1	0	1	
33	Goal framing theory	2	0	0	0	2	
34	Hofstede's cultural model	2	0	1	0	1	
35	interdependence theory	2	0	2	0	0	
36	Ipsative Theory of Behaviour	2	0	2	0	0	
37	Model of Material Possession	2	0	2	0	0	
38	Moral licensing	2	0	0	0	2	
39	New Ecological Paradigm	2	0	1	0	1	
40	Protection motivation theory	2	0	0	1	1	
41	Rational choice theory	2	0	2	0	0	
42	Reactance theory	2	1	0	0	1	
43	Safe Routes to School 6E model	2	0	2	0	0	
44	Signalling theory	2	0	1	0	1	
45	Social representations theory	2	0	0	0	2	

4. REFERENCES

Berrang-Ford L, Pearce T, Ford JD (2015) Systematic review approaches for climate change adaptation research. Reg Environ Change 15:755–769. https://doi.org/10.1007/s10113-014-0708-7

Kellermeyer L, Harnke B, Knight S (2018) Covidence and Rayyan. jmla 106:. https://doi.org/10.5195/jmla.2018.513

McKeown S, Mir ZM (2021) Considerations for conducting systematic reviews: evaluating the performance of different methods for de-duplicating references. Syst Rev 10:38. https://doi.org/10.1186/s13643-021-01583-y

Sherman M, Berrang-Ford L, Lwasa S, et al (2016) Drawing the line between adaptation and development: a systematic literature review of planned adaptation in developing countries. WIREs Clim Change 7:707–726. https://doi.org/10.1002/wcc.416

Siders AR (2019) Adaptive capacity to climate change: A synthesis of concepts, methods, and findings in a fragmented field. WIREs Clim Change 10:. https://doi.org/10.1002/wcc.573

Tenzing JD (2020) Integrating social protection and climate change adaptation: A review. WIREs Clim Change 11:. https://doi.org/10.1002/wcc.626
